# MRgFUS for Uterine Myomas: Safety, Effectiveness and Pathogenesis

**DOI:** 10.1186/2050-5736-2-S1-A1

**Published:** 2014-12-10

**Authors:** Julia Kurashvili, Alexander Stepanov, Elena Kulabuchova, Olga Batarshina

**Affiliations:** 1 Research Centre of Obstetrics / Gynecology & Perinatology, Moscow, Russia

## Purpose

Myomas are common benign tumors in woman. Patients prefer non-invasive treatment to preserve fertility and avoid surgery. In 2004 FDA approved MRgFUS for uterine myoma. The current study examined the safety and efficacy of MRgFUS for different myomas characteristics. Methods and Materials: A total of 925 patients, aged 20-55, with symptomatic myomas underwent MRgFUS (2006-2012). Prior to the treatment patients underwent gynecological examinations, MRI, biopsy. Safety was determined by tracking adverse events. Efficacy was assessed by measurement of NPV. MRI with contrast enhancement, symptom scores, treatments durability were also used as efficacy measures.

## Results

There was a low rate of complications (0.6%). MRgFUS resulted in volume reduction, symptomatic improvement, increase in the QoL, reduction in the vascularity, and long-term durability (4.5-5 years). A durability of symptoms improvement correlated with an NPV. MRgFUS was more effective for patients with less than 3 myomas unobstructed, myomas characterized by MR-hypo-intensity, a diameter of 2 to 6-8 cm, and an intramural component greater than 30%. Hypo-intense myomas are optimal candidates because the abundant connective tissue absorbs the FUS energy. Mitotically active and edematous myomas are not ideal due to limited connective tissue and the absence of a substratum for FUS absorption, respectively.

## Conclusion

MRgFUS is a safe method of symptomatic treatment, effective in prevention of clinical symptoms, preparation for transcervical myomectomy, delay of surgery.

